# Evaluation of Chinese HIV Mobile Apps by Researchers and Patients With HIV: Quality Evaluation Study

**DOI:** 10.2196/52573

**Published:** 2024-01-26

**Authors:** Peng Liu, Lingmeng Wang, Fuzhi Wang

**Affiliations:** 1 School of Health Management Bengbu Medical College Bengbu China; 2 Innovation Team of Health Information Management and Application Research Bengbu Medical College Bengbu China

**Keywords:** HIV, mobile app, evaluation, mobile phone

## Abstract

**Background:**

Against the backdrop of globalization, China remains one of the most heavily burdened countries in Asia with regard to AIDS. However, many high-risk groups and patients affected by AIDS may be less likely to actively seek care from medical institutions because of fear of experiencing shame or discrimination. Mobile apps provide a promising avenue for supporting the prevention, diagnosis, and treatment of AIDS. However, a comprehensive systematic evaluation of these mobile apps’ functionality and quality has not been conducted yet.

**Objective:**

This study aims to identify the available mobile apps for AIDS in China, assess and discuss the functional features and quality of these Chinese AIDS mobile apps, and offer decision support for patients and clinical practitioners in accessing high-quality AIDS mobile apps. Furthermore, based on the evaluation results, recommendations for improvement will be provided.

**Methods:**

A systematic search was conducted on the Qimai app data platform, the Aladdin WeChat applet data platform, and WeChat to identify mobile apps related to AIDS. A snowball sampling method was used to supplement the potentially overlooked apps. The selected mobile apps underwent a rigorous screening process based on unified criteria. Subsequently, assessments were independently undertaken by 3 separate researchers and 2 patients with HIV, using both the Mobile App Rating Scale (MARS) and the User Mobile App Rating Scale (uMARS). Quantitative interpretations of the data were facilitated by the MedCalc statistical software (version 20.217, MedCalc Software).

**Results:**

A total of 2901 AIDS mobile apps were included in the study, with 2897 identified through information retrieval and an additional 4 added via snowball sampling. After a rigorous selection process, 21 apps were determined to be usable. Among them, the Hong Feng Wan app achieved the highest combined average score, calculated based on the MARS (3.96, SD 0.33) and uMARS (4.47, SD 0.26). Overall, there was no significant correlation between MARS and uMARS (*r*_app quality total score_=0.41; *P*=.07; *r*_subjective quality_=0.39; *P*=.08). A notable issue was the widespread lack of user privacy protection, with only 24% (5/21) of the apps offering this feature.

**Conclusions:**

The number of available Chinese AIDS mobile apps is limited, with WeChat applets dominating the market. Nonetheless, the performance of WeChat mini-apps is generally inferior to that of independent apps, and there may be significant discrepancies between assessments conducted by researchers and those provided by genuine end users, emphasizing the necessity of involving real users in the development and evaluation of HIV mobile apps. In addition, developers of these Chinese HIV mobile apps need to devote attention to improving privacy protection mechanisms, in addition to considering the evaluations of researchers and real users. This will help attract more users and increase user loyalty.

## Introduction

### Background

AIDS is a chronic infectious disease caused by HIV infection, resulting in high mortality rates. Since the first reported case of AIDS, it has rapidly spread worldwide, becoming a major public health concern. China, influenced by globalization, is no exception. According to the 2021 National Notifiable Infectious Diseases Summary released by the Chinese Center for Disease Control and Prevention, AIDS has the highest mortality rate and death toll, with 19,623 reported deaths. Available data indicate that as of 2022, the number of confirmed AIDS cases in China has exceeded 1.05 million and is projected to surpass 1.6 million infections by 2023. AIDS remains a significant infectious disease affecting public health and socioeconomic development in China [1].

For individuals who are at a high risk of HIV infection but have not yet been infected, pre-exposure prophylaxis (PrEP) is a highly effective method of preventing HIV through daily medication. However, the current use of and adherence to PrEP remain low. However, mobile apps have shown effectiveness in promoting PrEP use and adherence among high-risk populations [2]. Muessig et al [3] highlighted the advantages of internet and mobile-based interventions, which facilitate wider dissemination of PrEP at a lower cost compared with conventional methods. The MyChoices app developed by Biello KB demonstrated feasibility and potential in improving HIV PrEP use rates among gay and bisexual men in the United States [4]. The prevalence of HIV infections remains disproportionately high among gay, bisexual, and other men who have sex with men (GBMSM) [5]. With the widespread use and convenience of smartphones, sexual networking apps have replaced traditional dating websites as the primary online social platform for GBMSM. As such, apps have become crucial venues for sexual health research [6]. Targeted video and text-based sexually transmitted infection and HIV information provided through mobile apps has proven effective in reducing new infection rates among GBMSM [5].

For people living with HIV, the highly active antiretroviral therapy developed by scientist David Ho represents the most effective treatment method. Highly active antiretroviral therapy can suppress HIV replication and prevent the emergence of drug-resistant viruses. However, strict adherence is required, necessitating patients to adhere to prescribed medication schedules [7]. Despite simplified treatment regimens, adherence remains challenging for some people living with HIV [8]. Evidence suggests that HIV case management can improve treatment adherence and quality of life and reduce risky sexual behavior [9,10]. However, case managers face significant workloads and limited policy and funding support [11,12]. Mobile health (mHealth) apps have demonstrated potential in assisting people living with HIV with effective self-management and delivering personalized interventions. Schnall et al [13,14] identified ideal features for an HIV app, including reminders, health information delivery, medication logs, communication, settings, and search functions. However, comprehensive apps meeting these criteria are scarce in the current market, and there is a lack of rigorously evaluated mHealth apps specifically designed for people living with HIV [13,14]. Yang et al [15] found that most Chinese GBMSM apps, selected based on relaxed inclusion criteria, primarily focused on dating and lacked HIV prevention and health information.

For both high-risk populations and individuals infected with HIV, HIV testing plays a vital role in combating HIV. Early HIV testing allows individuals to learn about their infection status promptly, facilitating timely access to antiretroviral therapy and significantly reducing mortality rates. However, fear of shame or discrimination may discourage many high-risk populations and patients from actively seeking care at health care facilities [16-19]. With the increasing prevalence of mobile phones, various mHealth interventions have been developed to diversify HIV self-testing (HIVST) approaches, including telephone hotlines, SMS text messaging–based interventions, and internet-based interventions. These interventions have shown potential in improving testing rates, particularly among hard-to-reach populations [20-26]. Although these platforms have achieved varying levels of success, the use of mobile apps has emerged as a highly popular trend because of their flexibility and scalability. For instance, the mLab app serves to enhance users’ understanding of their HIV test results while facilitating their access to pertinent HIV information and services [27]. Another noteworthy app is Aspect HIVST, which offers an acceptable means of uploading mobile HIVST results and demographic information to a centralized database [28]. In addition, ApiDé serves as a multilingual electronic tool (app) that assists health care providers in offering and explaining HIV screening to immigrants facing language barriers [29].

In conclusion, the use of mobile apps is becoming increasingly prevalent in HIV/AIDS prevention and control. These apps provide a convenient and accessible means for high-risk individuals and patients to access information, consultations, and support related to HIV/AIDS. They serve as effective adjunct measures in improving antiretroviral therapy adherence and reducing AIDS incidence rates, thereby mitigating the currently imbalanced resource allocation between patients and health care providers involved in antiviral treatment. However, research on Chinese AIDS mobile medical apps is relatively scarce and late, and empirical studies evaluating the effectiveness of HIV mobile medical apps remain sparse. Therefore, the effectiveness of Chinese HIV mobile apps in meeting user needs requires further assessment. To evaluate the efficacy of AIDS mobile apps in China, the Mobile App Rating Scale (MARS) and the User Mobile App Rating Scale (uMARS) scales, which are widely accepted and applied for uses such as assessing chronic disease management [30], COVID-19 tracking [31], psychiatric interventions [32], physical exercise among older people [33], and menstrual monitoring [34], were incorporated into this study as standardized assessment tools for the quality of mobile apps.

### Objectives

This study aims to achieve the following objectives:

1. To identify a comprehensive list of available Chinese mobile apps for HIV/AIDS.
2. To evaluate the functional features and quality of Chinese HIV/AIDS mobile apps from 2 distinct standpoints: those of researchers and those of individuals diagnosed with HIV, and to provide decision support for AIDS-related groups and health care professionals in accessing high-quality AIDS mobile apps.
3. To conduct a thorough analysis of the evaluation results and to provide improvement recommendations based on the findings, with the ultimate goal of enhancing the quality of Chinese HIV/AIDS mobile apps.

## Methods

### Search Strategy

Considering the use status of Chinese AIDS apps, a systematic search was conducted on the Qimai app data platform, Aladdin WeChat applet data platform, and WeChat from February 18 to 19, 2023, adhering to the PRISMA (Preferred Reporting Items for Systematic Reviews and Meta-Analyses) guidelines. The specific search strategies are as follows.

#### Data Collection of Apps

The Qimai platform, a well-known domestic mobile app data analytics platform, was used to retrieve AIDS-related apps. Qimai provides comprehensive data on iOS and Android app markets (including Huawei, Baidu, Xiaomi, Vivo, etc), along with app store optimization and app store search marketing optimization service tools and SearchAds data reports. It also offers professional data analysis and optimization strategies. The platform features an intelligent keyword expansion and association tool, which proved valuable for obtaining the required data for this study. In this research, the Qimai Keyword Expansion Assistant was used to search for AIDS-related terms. We selected keywords with a relevance score exceeding 50% and a search index surpassing 4605 (typically indicating a higher search frequency) to identify relevant apps that met the criteria. The search was conducted on February 18, 2023.

#### WeChat Applet Data Collection

WeChat applets, launched in China in 2017, are app programs developed on the WeChat platform. These applets offer users the convenience of accessing and using various functionalities and services directly within the WeChat app without requiring any separate downloads or installations. Known for their lightweight, fast, and user-friendly nature, they have gained popularity. To collect WeChat applet data, searches were performed on both the Aladdin Index and WeChat. The Aladdin Index, developed by Beijing Aladdin Future Technology Co, Ltd, serves as a ranking platform for the WeChat applet, providing a comprehensive reference for applet developers’ operations nationwide. For this study, keywords such as *HIV*, *AIDS*, *Human Immunodeficiency Virus*, *Acquired Immunodeficiency Syndrome*, as well as their corresponding Chinese keywords with similar meanings, such as *huo-de-xing-mian-yi-que-xian-zong-he-zheng,* *ai-zi*, *ai-zi-bing*, and *ai-si-bing*, were used to search for the WeChat applet related to AIDS in both the Aladdin applet list and WeChat. The search was conducted on February 19, 2023.

#### Supplementary Method

In collaboration with the HIV prevention and control team at the local Centers for Disease Control and Prevention (CDC), a snowball sampling method was used to distribute electronic questionnaires to individuals living with AIDS during their follow-up visits conducted by the CDC staff. The questionnaires (without personal information and providing adequate privacy protection) aimed to inquire about the HIV/AIDS apps they had used. Participants were encouraged to provide feedback using the Wenjuanxing platform and to share the electronic questionnaire with other individuals living with AIDS they knew. This supplementary method aimed to identify AIDS-related apps that were widely used within the AIDS community but were not captured by the aforementioned search strategies. The feedback collection period was extended from February 20 to May 19.

The exclusion criteria for apps were as follows: (1) nonsimplified Chinese language, (2) games, (3) apps with excessively limited functionality, (4) mobile apps that did not primarily focus on HIV or AIDS-related content, (5) >50% of the content is inaccessible, (6) apps that had terminated their services, (7) apps restricted to internal use by specific personnel (apps had to be accessible and functional for evaluation purposes), and (8) apps predominantly intended for advertising and product sales.

### Evaluation Tool

In this study, we selected the MARS and uMARS scales as the evaluation tools for assessing the quality of the apps. These scales were chosen for 2 main reasons. First, these scales have been extensively validated and have demonstrated good reliability and validity across different contexts. Second, in our study, the included apps were a mix of independent apps and WeChat applets, with the majority being WeChat applets rather than independent apps. However, the MARS and uMARS scales offer broader applicability in this regard.

Developed by Zelmer et al [35], the MARS scale provides a standardized set of criteria for evaluating mobile apps in terms of engagement, functionality, esthetics, and information quality. The MARS scale has been widely used in user research, mobile app evaluations, and related fields. Previous studies have indicated that the MARS scale demonstrates good validity and reliability, making it a reliable measurement tool [30-34]. The uMARS scale, developed by Manning et al [36], serves as an extension of the original MARS scale, specifically designed to prioritize the assessments of average users toward mobile apps.

### Evaluation Process

The evaluation was conducted by 3 researchers. Rater 1 (PL) holds a master’s degree in medical informatics and has extensive experience in medical information analysis. Rater 2 (Bin Li) holds a master’s degree in computer science and has abundant software development experience. Rater 3 (FW), an esteemed scholar in medical informatics in China, possesses a master’s degree in computer science and a PhD in social medicine. Being an expert in app quality evaluation, rater 3 boasts rich experience and high authority in research on assessing mobile app quality. Both rater 1 and rater 2 were recruited as real patients with HIV. The evaluations by the researchers and patients with HIV were conducted separately to avoid any interference. Each group followed a 3-step evaluation process. First, basic information for 21 apps, including their names, developers, platforms, and core functionalities, was collected from the SevenMa platform, Aladdin WeChat platform, and WeChat. Second, 2 WeChat applets and 2 independent apps were randomly selected for pilot evaluation. Before conducting the pilot evaluation, all 3 raters watched training videos on the MARS scale to better understand the purpose and significance of each item on the scale. Third, the 21 collected apps underwent a formal evaluation. To ensure consistent evaluation results, rater 1 and rater 2 independently assessed the same samples, completing the evaluation of the nth subdimension for all samples before moving on to evaluate the n+1th dimension. In cases where rater 1 and rater 2 had conflicting evaluations, we applied the Delphi method, which involved multiple rounds of consultation and feedback with experts, to achieve agreement regarding the discrepancies. Rater 3, an expert with relevant qualifications, reassessed the dimension that exhibited disagreement. The evaluation results from rater 3 were then shared with rater 1 and rater 2, along with appropriate explanations. This gave rater 1 and rater 2 the opportunity to revise their ratings and evaluations. If substantial differences persisted even after re-evaluation, the entire process was repeated until their evaluations converged or reached a satisfactory level of agreement.

### Statistical Analysis

All analyses were performed using MedCalc software (version 20.217, MedCalc Software). Descriptive scores were derived from the MARS and uMARS scales. To evaluate the reliability of the raters’ assessments, Bland-Altman analysis was used to assess both interrater agreement and the range of variability in their scores. Visual representations were also used to visualize the differences in ratings between the 2 evaluators, providing a more concrete quantification of the discrepancies.

### Ethical Considerations

This study focused solely on mobile apps and did not involve the collection of any personal information or data. Therefore, there were no ethical concerns or disputes associated with this research.

## Results

### Overview

**Figure 1:**
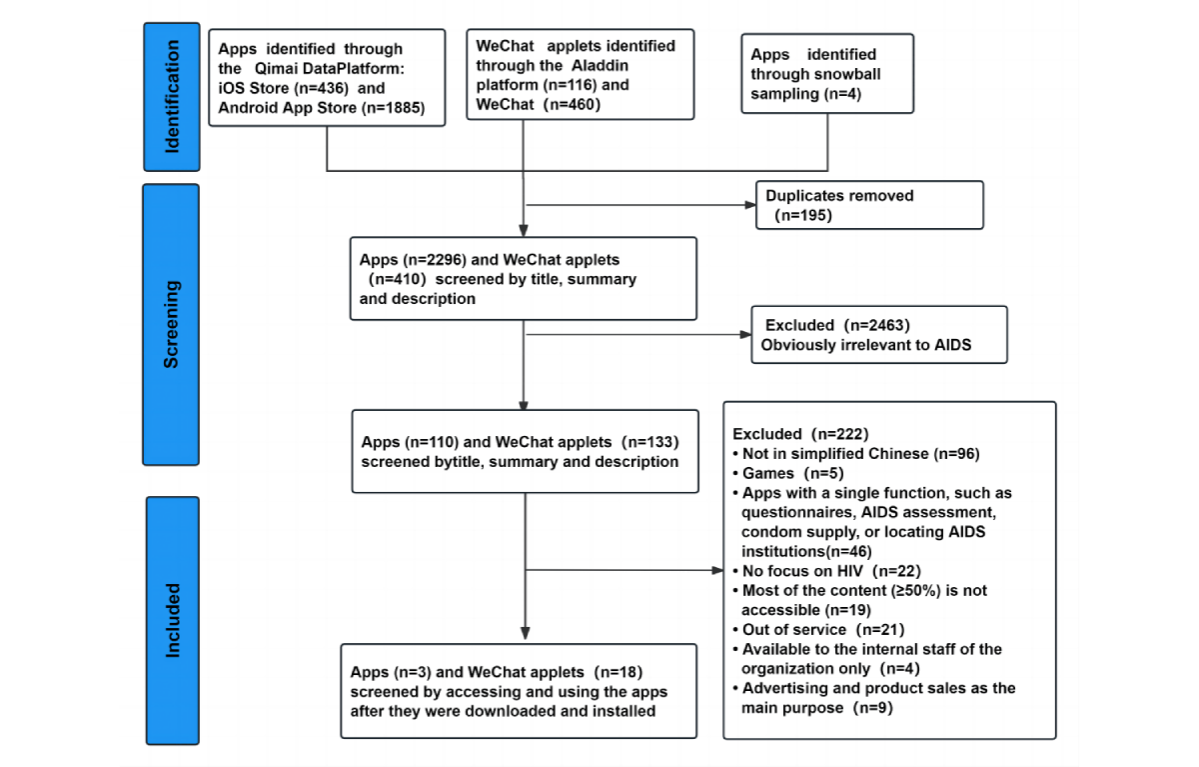
PRISMA (Preferred Reporting Items for Systematic Reviews and Meta-Analyses) flowchart of the selection process for inclusion of the apps.

**Figure 2:**
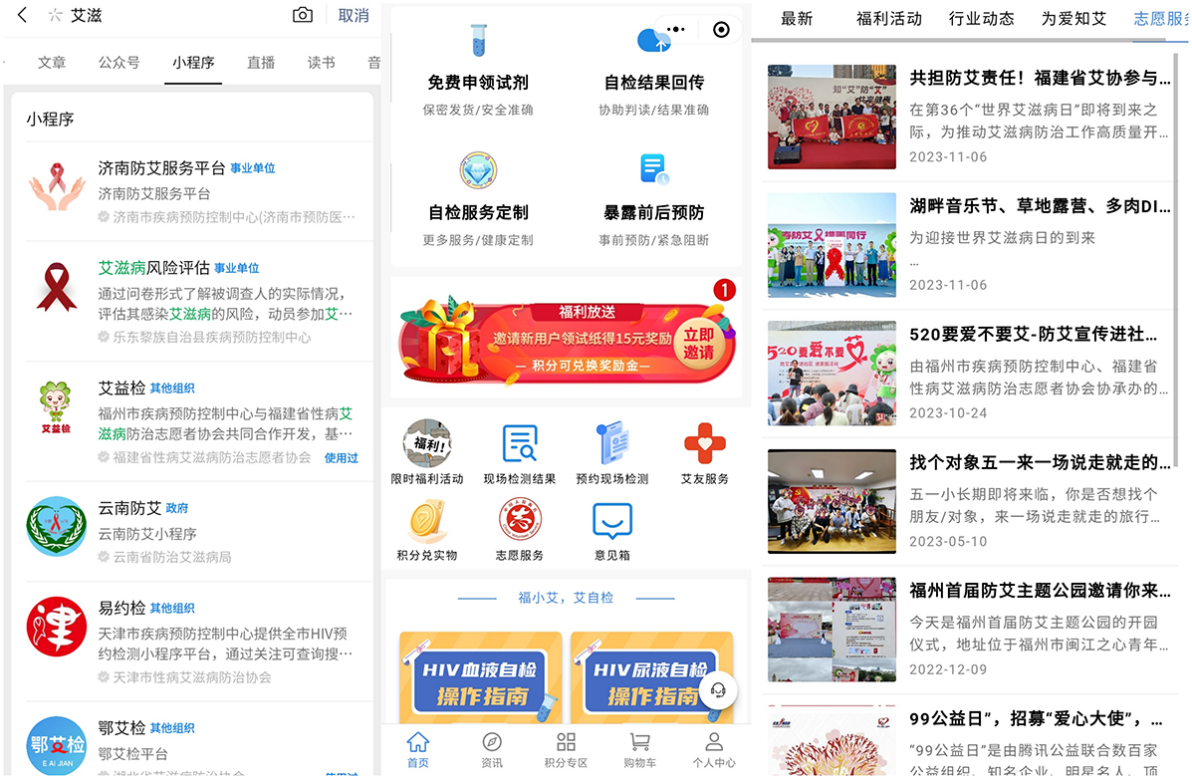
Example of a typical Chinese AIDS mobile app.

A total of 21 apps that met the criteria were obtained through screening, with 18 being WeChat applets, dominating in terms of quantity, whereas only 3 were independent apps (Figure 1). Representative AIDS mobile apps were also displayed, as detailed in Figure 2. The characteristics of these apps are shown in Tables 1 and 2.

The developers of these apps can be categorized into 3 groups: companies (6/21, 29%), government organizations (7/21, 33%), and nonprofit social organizations (8/21, 38%). In addition, of the 21 included apps, 18 (86%) provided HIV/AIDS knowledge dissemination, 12 (57%) offered appointment booking for testing, 11 (52%) had counseling services, 8 (38%) had features related to PrEP and postexposure prophylaxis (PEP), 2 (10%) had community networking functionalities, and only 1 (5%) had live streaming capabilities. Of the 21 apps included in the study, 16 (76%) were able to update their content within a year, whereas 5 (24%) had not been updated for >1 year. Only 6 apps had privacy protection features, all of which provided privacy policies or agreements, with 2 of the apps offering privacy password functionality.

**Table 1:** General characteristics of the AIDS mobile apps (N=21).

Features		Total apps, n (%)	Independent apps (n=3), n (%)	WeChat applets (n=18), n (%)
**Affiliation**				
	Company	6 (29)	3 (14)	3 (14)
	Government organization	7 (33)	0 (0)	7 (33)
	Nonprofit social organization	8 (38)	0 (0)	8 (38)
**Content type**				
	Live streaming	1 (5)	0 (0)	1 (5)
	Appointment for testing	15 (71)	3 (14)	12 (57)
	Consultation	10 (48)	2 (10)	8 (38)
	AIDS knowledge	18 (86)	2 (10)	16 (76)
	PrEP^a^ or PEP^b^	8 (38)	3 (14)	5 (24)
	Web community	2 (10)	1 (5)	1 (5)
**Last updated**				
	Within 1 month	5 (24)	1 (5)	4 (19)
	Within 2 to 6 months	8 (38)	2 (10)	6 (29)
	Within 7 to 12 months	3 (14)	0 (0)	3 (14)
	>1 year	5 (24)	0 (0)	5 (24)
**Privacy**				
	Privacy policy or agreement	6 (29)	3 (14)	3 (14)
	Privacy password	2 (10)	2 (10)	0 (0)

^a^PrEP: pre-exposure prophylaxis.

^b^PEP: postexposure prophylaxis.

**Table 2:** Basic information about the AIDS mobile apps.

App name	App OR WC^a^	Knowledge sharing	Appointment testing	Live streaming	Consultation	Risk assessments	PrEP^b^ and PEP^c^	Web-based community	Privacy protection
Chabei	WC	✓	✓		✓		✓		✓
Danlan Happy Test	WC	✓	✓						
Suzhou Red Ribbon	WC	✓			✓				✓
Baiyin HIV test	WC	✓	✓		✓		✓		
Linqu County CDC^d^	WC	✓	✓			✓			
Wu Ai Fang Hua	WC	✓						✓	
E Ai Jian	WC		✓		✓	✓	✓		
Ai Yi Jian	WC	✓	✓				✓		
Rong Ai Jian	WC	✓	✓		✓	✓			
Liaocheng Dongchangfu District Anti-AIDS Service Platform	WC	✓			✓	✓	✓		
Zhecheng County AIDS consulting and test	WC	✓	✓		✓	✓			
Qingai Health Services	WC	✓	✓		✓				
Ai Zhiku	WC	✓							
Ai Cheng Wang Shi	WC	✓	✓						
Beijing AIDS Association	WC		✓	✓					
Douai Check	WC	✓	✓			✓			
Nanyue Gaozhibao	WC	✓							
Red Ribbon Volunteer House	WC	✓							
Hong Feng Wan	App	✓	✓		✓		✓	✓	✓
Life4me+	App		✓				✓		✓
Xiao Ai	App	✓	✓		✓		✓		✓

^a^WC: WeChat applet.

^b^PrEP: pre-exposure prophylaxis.

^c^PEP: postexposure prophylaxis.

^d^CDC: Centers for Disease Control and Prevention.

### Functionality of the App

Most mobile apps (18/21, 86%) provided HIV/AIDS knowledge dissemination, and 71% (15/21) of the apps offered appointment booking for testing. Nearly half of the apps (10/21, 48%) provided counseling services, whereas 38% (8/21) of the apps offered features related to PrEP or PEP. Only 10% (2/21) of the apps had functionalities for live streaming and web-based community.

In addition to these functions related to function evaluation, we also included additional statistics on privacy protection settings. Among the 21 apps included in the study, only 6 (29%) provided privacy protection settings and 2 (10%) designed privacy passwords.

### Quality of the App

#### Overview of App Composite Scores

The composite scores for the app quality total score in MARS and uMARS were obtained by averaging the scores for each app. The overall composite score for the 21 included apps was 3.43 (mean_app quality total score of MARS_=3.47, SD 0.37; mean_app quality total score of uMARS_=3.38, SD 0.53). Hong Feng Wan achieved the highest composite score, with a score of 4.22 (mean_app quality total score of MARS_=3.96; mean_app quality total score of uMARS_=4.47), followed by Wu Ai Fang Hua with a composite score of 3.9 (mean_app quality total score of MARS_=4.06; mean_app quality total score of uMARS_=3.74), and Chabei secured the third rank with a composite score of 3.76 (mean_app quality total score of MARS_=3.66; mean_app quality total score of uMARS_=3.86). Suzhou Red Ribbon obtained the lowest composite score, with a score of 2.68 (mean_app quality total score of MARS_=3.67; mean_app quality total score of uMARS_=1.68).

#### Comparative Analysis of MARS Score and uMARS Score

**Figure 3:**
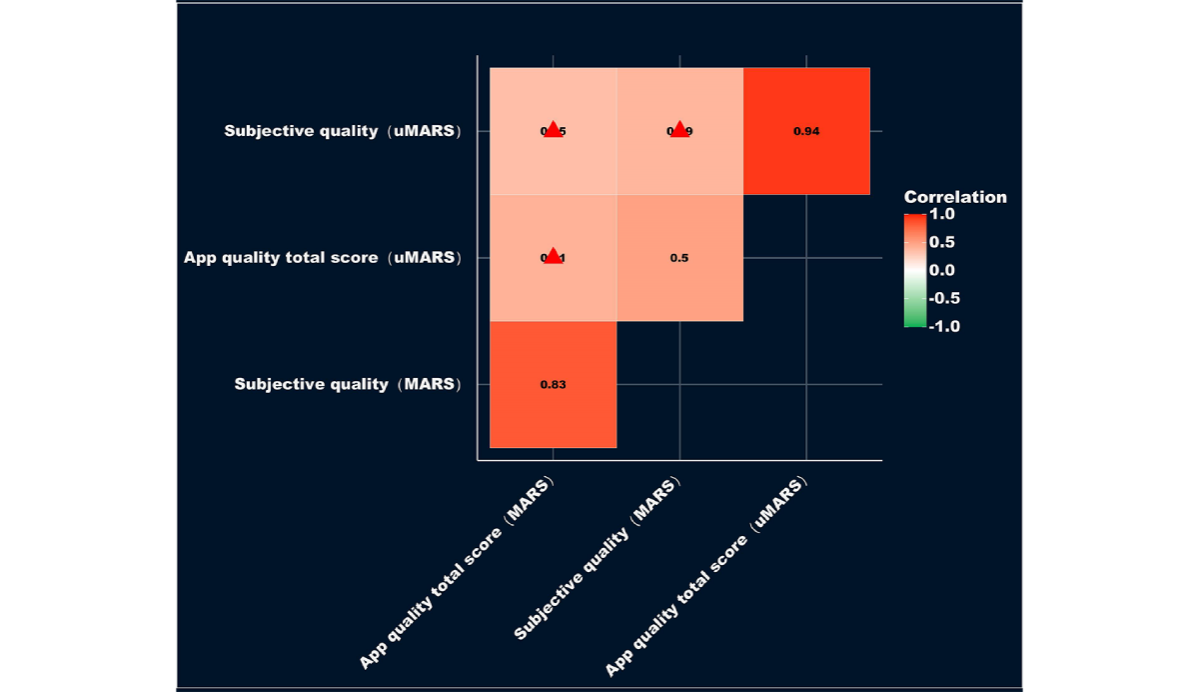
Results of correlation analysis between the Mobile App Rating Scale (MARS) and the User Mobile App Rating Scale (uMARS) scores.

Figure 3 presents a correlation analysis between the scores of MARS and uMARS. No significant relationship was observed between the app total quality scores of MARS and uMARS (0.41; *P*=.07) or between their subjective quality scores (0.39; *P*=.08). The app most reflective of the disparity between MARS and uMARS is Suzhou Red Ribbon, which had the greatest discrepancy between its app total quality scores on MARS (3.67) and uMARS (1.68). Its score on uMARS (1.68) was the lowest in the ranking, with respective dimensional scores of engagement (1.8), functionality (1.5), esthetics (1.67), information (1.75), and subjective quality (1). Conversely, for MARS, it ranked seventh for the app quality total score, with respective dimensional scores of engagement (2.9), functionality (4.38), esthetics (3.5), information (3.92), and subjective quality (2.5). There was considerable variation disclosed in the rankings of the most highly rated apps between MARS and uMARS. The top 5 app total quality scores on MARS were Wu Ai Fang Hua (4.06), Hong Feng Wan (3.96), Ai Yi Jian (3.95), Qing Ai Health Services (3.77), and Ai Zhiku (3.76), whereas the top 5 on uMARS were Hong Feng Wan (4.47), Chabei (3.86), Wu Ai Fang Hua (3.74), Linqu County CDC (3.66), and Zhecheng County AIDS consulting and test (3.62).

**Figure 4:**
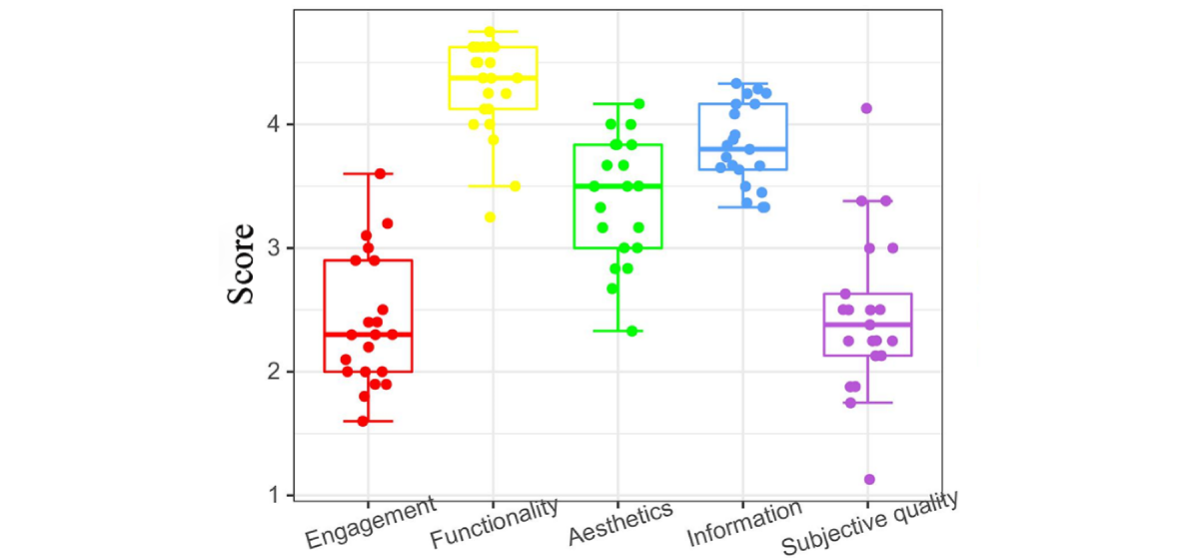
Box plot of the Mobile App Rating Scale (MARS) score.

**Figure 5:**
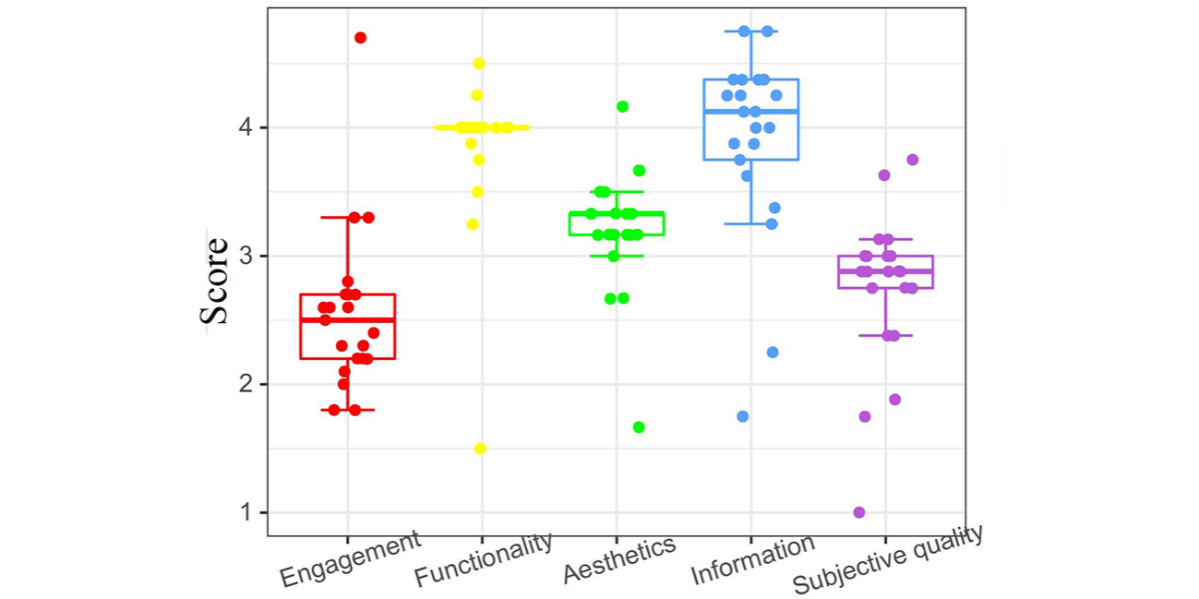
Box plot of the User Mobile App Rating Scale (uMARS) score.

Interpreting composite data from Figures 4 and 5 and Table 3 reveal that within the 4 MARS scale dimensions of the evaluated 21 apps, the functionality dimension achieved the highest score (4.28, SD 0.39).

The information (mean 3.82, SD 0.33), aesthetics (mean 3.4, SD 0.49), and engagement (mean 2.4, SD 0.53) dimensions sequentially trailed behind. In contrast, under the uMARS scale’s evaluation, the information dimension prevailed with the top score (mean 3.89, SD 0.74), followed by the functionality (mean 3.84, SD 0.59), aesthetics (mean 3.21, SD 0.48), and engagement (mean 2.56, SD 0.63) dimensions.

**Table 3:** The MARS^a^ and uMARS^b^ scales’ scores for apps. The top 5 apps with app quality total score for each dimension are italicized.

App name	App quality ratings																
	Section A: engagement			Section B: functionality			Section C: esthetics			Section D: information			App quality total score			Section E: subjective quality	
	MARS	uMARS	MARS		uMARS	MARS		uMARS	MARS		uMARS	MARS		uMARS	MARS		uMARS

Ai Cheng Wang Shi	2.00	2.10	4.25		4.00	2.33		3.33	3.50		3.88	3.02		3.33	1.88		2.75
Ai Yi Jian^c^	*3.00*	2.80	*4.63*		4.00	*4.00*		3.17	*4.17*		4.13	*3.95*		3.52	3.38		2.75
Ai Zhiku^c^	*2.50*	2.30	*4.38*		4.00	*4.00*		3.17	*4.17*		4.38	*3.76*		3.46	3.00		3.00
Baiyin HIV test	2.30	2.60	4.63		4.00	3.50		3.33	3.67		4.00	3.52		3.48	2.50		2.88
Beijing AIDS Association	2.40	2.20	3.50		4.00	3.33		3.17	3.65		4.25	3.22		3.40	2.50		2.88
Chabei^d^	2.20	*2.70*	4.50		*4.50*	3.67		*3.50*	4.29		*4.75*	3.66		*3.86*	3.00		3.75
Danlan Happy Test	1.60	2.30	3.25		3.75	2.84		3.00	3.33		2.25	2.63		2.83	1.13		2.38
Dou Ai Jian	1.90	1.80	4.13		3.25	3.00		2.67	3.45		3.25	3.12		2.74	2.25		1.75
E Ai Jian	2.30	2.70	4.75		4.00	3.84		3.33	3.74		4.38	3.66		3.60	2.25		3.00
Red Ribbon Volunteer House	2.00	2.50	3.88		4.00	3.00		3.17	3.37		4.13	3.06		3.45	2.13		2.88
Liaocheng Dongchangfu District Anti-AIDS Service Platform	2.00	2.20	4.50		4.00	3.50		3.33	4.25		4.25	3.56		3.45	2.25		2.75
Linqu County CDC^d^	2.10	*2.60*	4.63		*4.00*	3.17		*3.67*	3.80		*4.38*	3.42		*3.66*	2.50		3.00
Nanyue Gaozhibao	1.90	2.20	4.00		4.00	2.84		3.17	3.67		3.75	3.10		3.28	1.75		2.88
Qing Ai Health Services^c^	*2.90*	3.30	*4.50*		4.00	*3.84*		3.17	*3.83*		3.88	*3.77*		3.59	2.63		2.88
Rong Ai Jian	2.30	2.40	4.63		3.88	3.84		3.33	3.33		3.38	3.52		3.25	2.13		2.38
Suzhou Red ribbon	2.90	1.80	4.38		1.50	3.50		1.67	3.92		1.75	3.67		1.68	2.50		1.00
Wu Ai Fang Hua^c,d^	*3.20*	*3.30*	*4.63*		*4.00*	*4.17*		*3.67*	*4.25*		*4.00*	*4.06*		*3.74*	3.38		3.13
Zhecheng County AIDS consulting and test^d^	2.40	*2.60*	4.00		*4.00*	3.17		*3.50*	4.09		*4.38*	3.41		*3.62*	1.88		3.00
Life4me+	1.80	2.00	4.13		3.50	2.67		2.67	3.64		3.63	3.06		2.95	2.25		1.88
Hong Feng Wan^c,d^	*3.60*	*4.70*	*4.25*		*4.25*	*3.67*		*4.17*	*4.33*		*4.75*	*3.96*		*4.47*	4.13		3.63
Xiao Ai	3.10	2.70	4.38		4.00	3.50		3.33	3.88		4.25	3.71		3.57	2.38		3.13

^a^MARS: Mobile App Rating Scale.

^b^uMARS: User Mobile App Rating Scale.

^c^The top 5 apps in terms of app quality total score in MARS.

^d^The top 5 apps in terms of app quality total score in uMARS.

#### Comparative Analysis of Quality Between WeChat Mini-Apps and Independent Apps

As shown in Table 4, insignificant disparities are discernible between WeChat mini-apps and stand-alone apps regarding 2 metric domains: app quality (*P*_MARS_=.70; *P*_uMARS_=.54) and subjective app quality (*P*_MARS_=.48; *P*_uMARS_=.80).

**Table 4:** Independent samples *t* test for unequal variances.

MARS^a^ quality	MARS, mean (SD)	uMARS^b^, mean (SD)	MARS, *t* value (*df*)	uMARS, *t* value (*df*)	MARS, *P* value	uMARS, *P* value
App quality	—^c^	—	−0.448 (2.4)	0.73 (2.3)	.70	.54
WeChat applets	3.45 (0.37)	3.33 (0.50)	—	—	—	—
Independent apps	3.58 (0.47)	3.66 (0.76)	—	—	—	—
App subjective quality	—	—	−0.852 (2.2)	0.289 (2.3)	.48	.80
WeChat applets	2.39 (0.57)	2.72 (0.58)	—	—	—	—
Independent apps	2.92 (1.05)	2.88 (0.90)	—	—	—	—

^a^MARS: Mobile App Rating Scale.

^b^uMARS: User Mobile App Rating Scale.

^c^Not available.

#### Internal Consistency and Reliability Testing for MARS and uMARS Scores

**Figure 6:**
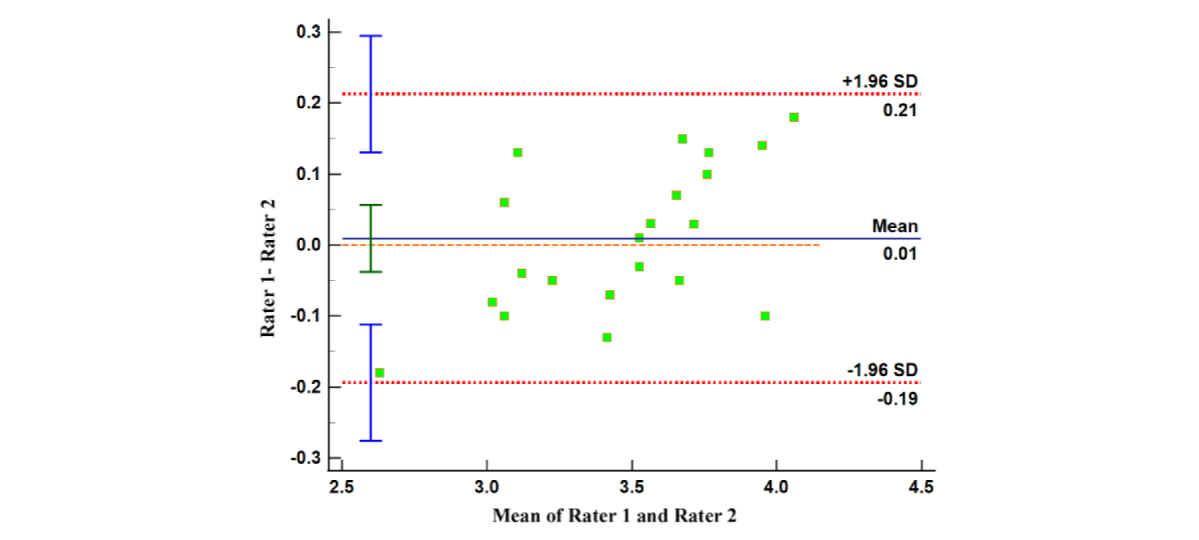
Bland-Altman plot of the app quality (Mobile App Rating Scale).

**Figure 7:**
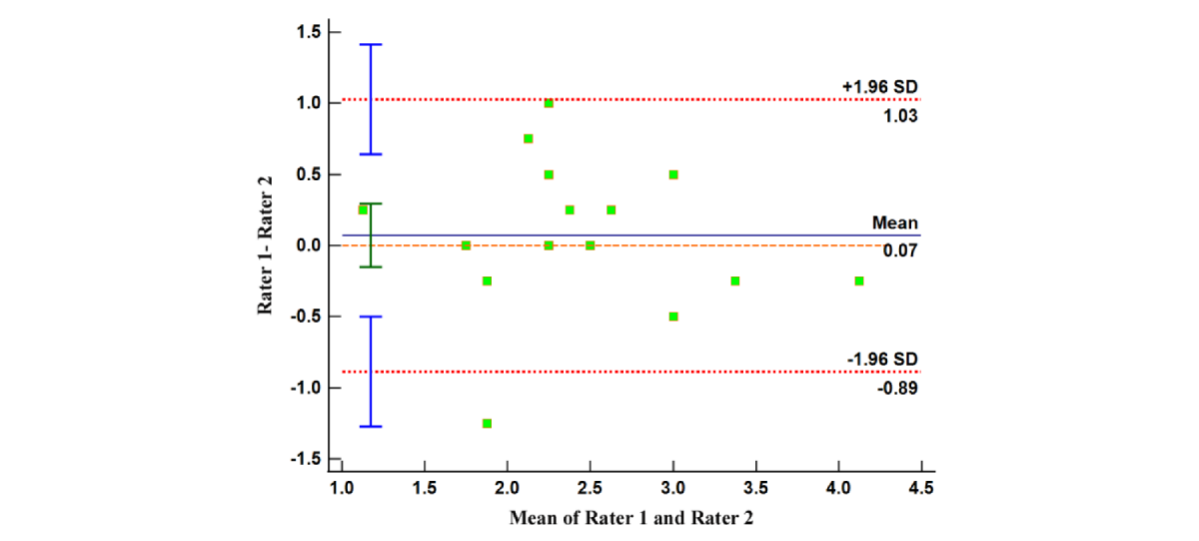
Bland-Altman plot of the app subjective quality (Mobile App Rating Scale).

**Figure 8:**
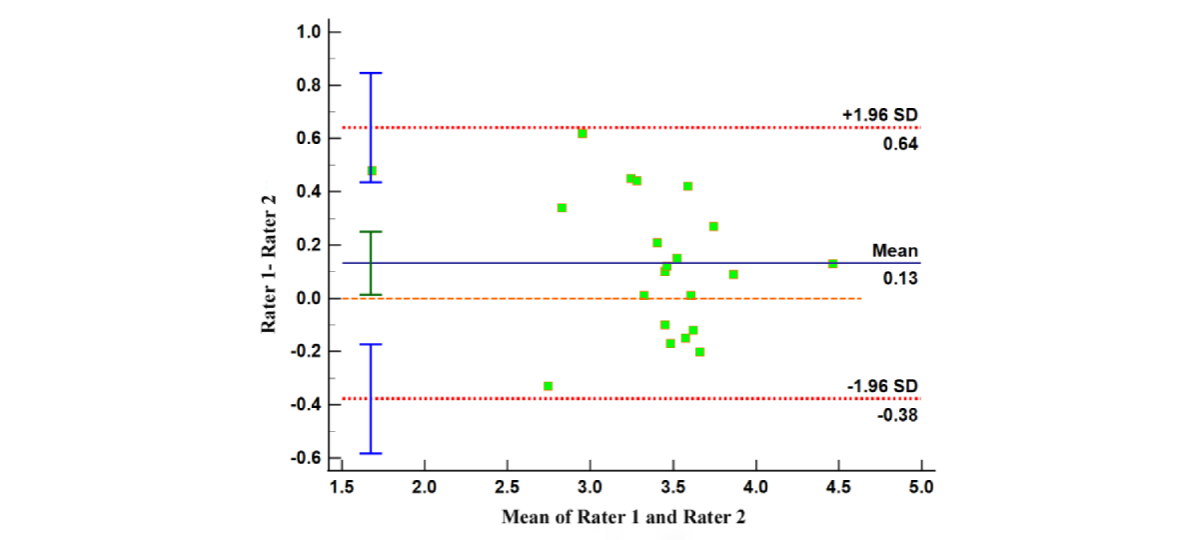
Bland-Altman plot of the app quality (User Mobile App Rating Scale).

**Figure 9:**
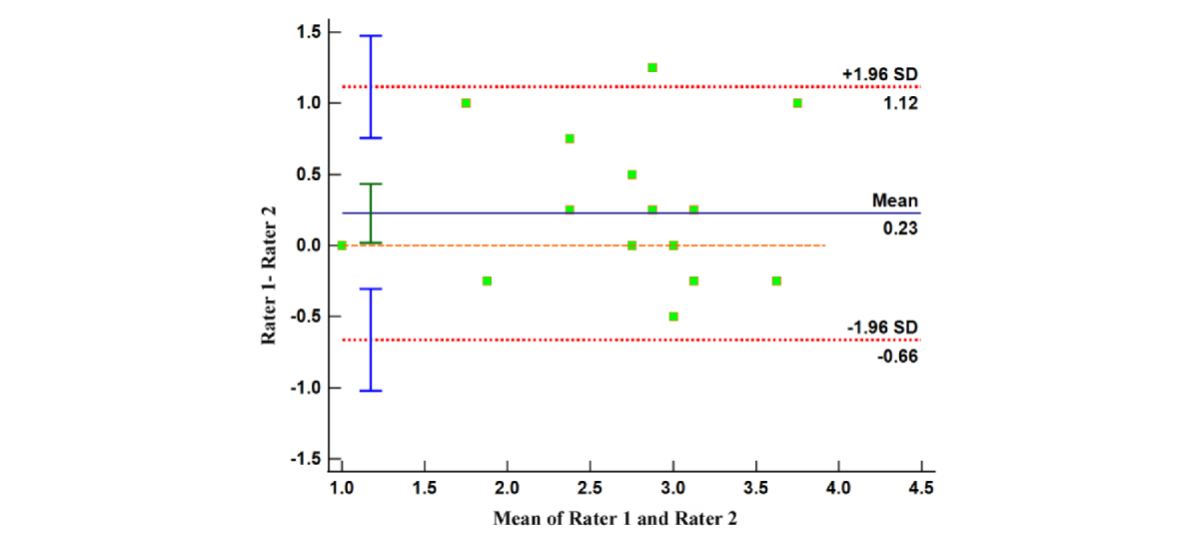
Bland-Altman plot of the app subjective quality (User Mobile App Rating Scale).

Similarly, we formed 4 Bland-Altman plots using the disparities and mean values between the scorings by reviewers on app quality (both MARS and uMARS) and app subjective quality (MARS and uMARS). The relatively limited range of the 95% limits of agreements suggest that the evaluators’ judgment outputs contain minor dissimilarities. A significant proportion of dots in all 4 Bland-Altman plots lie within the concordance interval (Figures 6-9), with their arithmetic means impressionably approaching 0 (Table 5).

This provides evidence of the high degree of internal uniformity and dependability in both MARS and uMARS scores.

**Table 5:** Summary of results of Bland-Altman analysis.

Index	95% LoA^a^	95% CI (arithmetic mean)	95% CI (LoA: upper limit)	95% CI (LoA: lower limit)
App quality (MARS^b^)	−0.19 to 0.21	−0.04 to 0.06	0.13 to 0.30	−0.28 to −0.11
App subjective quality (MARS)	−0.89 to 1.03	−0.15 to 0.29	0.64 to 1.41	−1.27 to −0.50
App quality (uMARS^c^)	−0.38 to 0.64	0.01 to 0.25	0.44 to 0.85	−0.58 to −0.17
App subjective quality (uMARS)	−0.66 to 1.12	0.02 to 0.43	0.76 to 1.47	−1.02 to −0.30

^a^LoA: limits of agreement.

^b^MARS: Mobile App Rating Scale.

^c^uMARS: User Mobile App Rating Scale.

## Discussion

### Principal Findings

This study provides a comprehensive statistical summary of the functionalities and characteristics of 21 Chinese HIV-related mobile apps, along with their quality assessment using the MARS scale. The primary objectives behind the development of these mobile apps were the dissemination of HIV prevention knowledge (18/21, 86%), appointment booking for testing (15/21, 71%), and counseling services (10/21, 48%). These mobile apps aim to assist users in HIV prevention and treatment while improving the quality of communication and interaction between patients and health care providers.

### Overview of App Functions

In recent years, the Chinese government has implemented a series of regulations and policies for HIV/AIDS prevention and has established an effective mechanism for HIV/AIDS control. These efforts have achieved certain progress in HIV/AIDS prevention. However, the burden of AIDS disease continues to increase, and the prevention and control situation remains severe. With the widespread adoption of smartphones and the rapid development of mobile internet, mobile apps have played an increasingly important role in disseminating HIV/AIDS knowledge and raising public awareness of prevention [1,37]. Among the 21 apps included in this study, 18 (86%) had HIV/AIDS knowledge dissemination functionality. This indicates that HIV/AIDS knowledge dissemination is an almost indispensable feature of Chinese HIV/AIDS mobile apps, which have become important tools for promoting HIV/AIDS knowledge in China.

In this study, 17 (81%) of the 21 mobile apps were identified, including 14 (67%) WeChat applets (such as Chabei, Danlan Happy Test, Baiyin HIV test, Suzhou Red Ribbon, Linqu County CDC, E Ai Jian, Ai Yi Jian, Rong Ai Jian, Liaocheng Dongchangfu District Anti-AIDS Service Platform, Zhecheng County AIDS consulting and test, Qing Ai Health Service, Ai Cheng Wang Shi, Beijing AIDS Association, and Dou Ai Jian) and 3 (14%) independent apps (Life4me+, Hong Feng Wan, and Xiao Ai). These apps provided various forms of health services, including appointment booking, counseling, risk assessment, and PrEP or PEP. A total of 2 mobile apps (Wu Ai Fang Hua and Hong Feng Wan) offered web-based community functionalities and scored excellently in terms of app quality and subjective quality, ranking among the top 2 positions. This finding is consistent with that of a previous study by Nour et al [38]. Therefore, we recommend considering the incorporation of web-based community functions when developing and designing mobile apps related to AIDS. This can encourage users to share their experiences, ask questions, provide suggestions, and offer mutual assistance and support. Organizing regular web-based or offline activities can also promote face-to-face interactions among users, thereby enhancing their sense of belonging and engagement.

Privacy protection is another crucial topic that should be given sufficient attention in AIDS-related mobile apps. As social stigmatization and discrimination are still associated with AIDS, many users are concerned about their personal information being disclosed or misused, posing a threat to their privacy and security [39]. Therefore, unlike general mobile apps, ensuring user privacy protection in AIDS mobile apps is of utmost importance, aligning with previous research findings [40]. However, in this study, of the 21 apps, only 5 (24%) mobile apps, including 2 (10%) WeChat applets (Chabei and Su Zhou Red Ribbon) and 3 (14%) apps (My Life+, Hong Feng Wan, and Xiao Ai), provided privacy policies or agreements. The average subjective quality score (mean 2.85, SD 0.77) was significantly higher than that of the mobile apps without privacy protection features (mean 2.34, SD 0.58). This observed phenomenon may be attributed to the fact that inadequate privacy protection design may lead to user wariness and lower intentions and frequencies of use regarding AIDS mobile apps [41]. Therefore, we strongly suggest that all AIDS-related mobile apps incorporate privacy policies or agreements to alleviate user concerns about privacy protection, enhance user trust, and promote willingness to use these apps. In addition to transparent privacy policies and user agreements, user education is also critical. Developers of AIDS mobile apps should provide users with relevant information and educational resources on privacy protection. This can be achieved through in-app prompts, tutorials, and frequently asked questions. Users should receive clear instructions on configuring their privacy settings, handling their personal data, and the steps to take in reporting privacy concerns or data breaches. These instructions should be easily accessible, comprehensible, and provide specific examples to guide users through each process.

### Rating of App Quality

According to the results from MARS scores, among the 4 dimensions of app quality, both researchers and patients with HIV rated 21 mobile apps lowest on engagement (mean_MARS_ 2.4, SD 0.53; mean_uMARS_ 2.56, SD 0.63), especially the WeChat mini-apps, which had the lowest average scores (mean_MARS_ 2.33, SD 0.43; mean_uMARS_ 2.47, SD 0.42). The average engagement scores for the 7 mobile apps developed by the Chinese government were even lower (mean_MARS_ 2.27, SD 0.33; mean_uMARS_ 2.34, SD 0.30). This underlined the concern that the level of engagement would remain low, even for officially developed apps. An analysis of the respective scores for each item within the MARS and uMARS engagement portions revealed a predominant lack of interest and amusement within these mobile apps, thus leading to decreased user engagement. Many AIDS-related mobile apps merely provide basic information and functions without interactive and stimulating designs to attract user participation, lacking a strong appeal to users. In addition, the absence of personalized and customized features limits user engagement. A study has shown a significant correlation between user engagement and an increase in the adoption rate of mobile apps [42]. Therefore, it is recommended that developers not only provide high-quality HIV/AIDS prevention and treatment information but also focus on meeting users’ needs in terms of multidimensionality, functionality, and depth. In addition, attention should be paid to design in terms of amusement, entertainment, customization, interactivity, and other participatory aspects. This will help attract more user participation and enhance user stickiness.

This investigation revealed a lack of correlation between the MARS and uMARS scores. Specifically, (1) the app with the lowest overall uMARS scores across all dimensions surprisingly ranked seventh in terms of MARS scores. (2) A notable discrepancy was found in the functionality dimension ratings among patients with HIV and researchers, exposing the highest demographic variance in this attribute. (3) Moreover, the apps predominantly preferred by patients with HIV exhibited robust performance in functionality and information dimensions, with the latter appearing particularly predominant. However, the apps gravitating toward researchers demonstrated high competence in functionality, esthetics, and information, with functionality being the most superior. These data imply possible significant divergences distinct between the evaluations of researchers and genuine users of the apps. To maintain superior app quality and consumer satisfaction, rigorous surveillance of app quality should be sustained from both research and real-user perspectives. This bifocal assessment permits the accurate identification of genuine user requirements and researcher appraisal, providing valuable insights for the pinpoint and scientific enhancement of both app quality and user experience.

In this study, we observed that most Chinese AIDS-related mobile apps are WeChat applets. A reason for this is that the WeChat applet has advantages such as not requiring downloading or installation, having minimal resource consumption, and high user retention rates, which independent apps do not possess [43]. Another reason is that the HIV/AIDS population is relatively niche [44], which means that the market for AIDS mobile apps represents a low-frequency demand niche market. Independent apps often overlook this niche market owing to their high development costs, whereas WeChat applets, with their low development costs and the ability to cater to low-frequency demand niches through segmented scene construction, can effectively meet the needs of this market. Although WeChat mini-apps dominate the landscape of mobile apps for HIV/AIDS in China, their comprehensive performance lags behind that of stand-alone apps. The app quality score (mean_MARS_ 3.45, SD 0.37 vs 3.58, SD 0.47; mean_uMARS_ 3.33, SD 0.50 vs 3.66, SD 0.76) and subjective quality score (mean_MARS_ 2.39, SD 0.57 vs 2.92, SD 1.05; mean_uMARS_ 2.72, SD 0.58 vs 2.88, SD 0.90) for WeChat mini-apps are both lower compared with stand-alone apps. A granular analysis of the scores in different dimensions reveals the greatest discrepancy in the engagement dimension, with WeChat mini-apps scoring markedly lower (mean_MARS_ 2.33, SD 0.43 vs 2.83, SD 0.93; mean_uMARS_ 2.47, SD 0.42 vs 3.13, SD 1.40). This can be attributed to the constraints and limitations imposed by the WeChat platform, which prevent WeChat applets from providing the same user experience as independent apps in terms of interface design, interaction methods, and customization settings. Improvement in these aspects should be a key focus for the future development of AIDS-related WeChat applets.

### Limitations

The mobile apps evaluated in this study represent a snapshot of the current status of Chinese HIV-related mobile apps during the research period. Over time, mobile apps may be removed or updated, so the list of included mobile apps meeting the criteria may change in the future. In addition, some mobile apps intended solely for internal organizational use or no longer available in the market were excluded.

### Conclusions

This study presents a systematic introduction to the functionality and quality of the currently available Chinese mobile apps for AIDS, providing valuable decision-making support for AIDS-related groups and health care professionals in accessing high-quality AIDS mobile apps. Through our systematic search and evaluation of existing Chinese mobile apps for AIDS, it has been observed that because of the lower demand frequency of AIDS mobile apps in China, less costly WeChat mini-apps have become the primary mode of app, and the overall quality attains a merely average level. A significant implication of our research is identifying the potentially significant discrepancy between the assessments made by researchers and the authentic users of the apps. Consequently, the inclusion of genuine users during the assessment and refinement stages of HIV apps is crucial. The main purpose of developing these mobile apps is to spread HIV prevention knowledge and facilitate booking appointments for testing and counseling services. However, most of these apps lack privacy protection features. Unlike general mobile apps, privacy protection is especially crucial in AIDS-related mobile apps because it directly affects users’ willingness to use them [40]. Therefore, the introduction of legal and ethical frameworks for privacy protection as well as privacy protection technologies is essential. In addition, enhancing user education on privacy protection and ensuring informed consent is of utmost importance. Research related to privacy protection in Chinese AIDS mobile apps may be a vital and urgent topic to address in the future. It is our intent that the findings of our research may function as a road map and reference for the future development of HIV apps in China. Furthermore, we aim to provide crucial decision-making support for individuals living with HIV in their quest for superior HIV apps.

## Abbreviations

CDC: Centers for Disease Control and Prevention
GBMSM: gay, bisexual, and other men who have sex with men
HIVST: HIV self-testing
MARS: Mobile App Rating Scale
mHealth: mobile health
PEP: postexposure prophylaxis
PrEP: pre-exposure prophylaxis
PRISMA: Preferred Reporting Items for Systematic Reviews and Meta-Analyses
uMARS: User Mobile App Rating Scale

## Data Availability

The data sets generated during and/or analyzed during this study are available from the corresponding author on reasonable request.

## References

[1] Wu Z, Chen J, Scott SR, McGoogan JM (2019). History of the HIV epidemic in China. Curr HIV/AIDS Rep.

[2] Sharpe JD, Kamara MT (2018). A systematic evaluation of mobile apps to improve the uptake of and adherence to HIV pre-exposure prophylaxis. Sex Health.

[3] Muessig KE, Nekkanti M, Bauermeister J, Bull S, Hightow-Weidman LB (2015). A systematic review of recent smartphone, internet and web 2.0 interventions to address the HIV continuum of care. Curr HIV/AIDS Rep.

[4] Biello KB, Hill-Rorie J, Valente PK, Futterman D, Sullivan PS, Hightow-Weidman L, Muessig K, Dormitzer J, Mimiaga MJ, Mayer KH (2021). Development and evaluation of a mobile app designed to increase HIV testing and pre-exposure prophylaxis use among young men who have sex with men in the united states: open pilot trial. J Med Internet Res.

[5] Downing Jr MJ, Wiatrek SE, Zahn RJ, Mansergh G, Olansky E, Gelaude D, Sullivan PS, Stephenson R, Siegler AJ, Bauermeister J, Horvath KJ, Chiasson MA, Yoon IS, Houang ST, Hernandez AJ, Hirshfield S (2023). Video selection and assessment for an app-based HIV prevention messaging intervention: formative research. Mhealth.

[6] Grov C, Stief M, Westmoreland DA, MacCrate C, Mirzayi C, Nash D, Together 5000 Study Team (2020). Maximizing response rates to ads for free at-home HIV testing on a men-for-men geosocial sexual networking app: lessons learned and implications for researchers and providers. Health Educ Behav.

[7] Di Mascio M, Markowitz M, Louie M, Hogan C, Hurley A, Chung C, Ho DD, Perelson AS (2003). Viral blip dynamics during highly active antiretroviral therapy. J Virol.

[8] Beauchemin M, Gradilla M, Baik D, Cho H, Schnall R (2019). A multi-step usability evaluation of a self-management app to support medication adherence in persons living with HIV. Int J Med Inform.

[9] Yang LW, Renfang NI, Xiulan W (2016). The measures and effects evaluation of case management in HIV-infected women during perinatal period. Adv Nurs Pract.

[10] Wen YL, Xian-yu Y, Jing-qiu F (2018). Influence of case intervention on behaviors of homosexual behavior patients with HIV infection. Chin J Nosocomiol.

[11] Tian Bo LJ, Kun-li W, Rong H (2018). Exploration of case management mode in antiretroviral therapy. J Dermatol Venereol.

[12] Kong F (2018). WH-q: research progress for nursing interventions for HIV/AIDS patients. Chinese Gen Pract Nurs.

[13] Schnall R, Mosley JP, Iribarren SJ, Bakken S, Carballo-Diéguez A, Brown Iii W (2015). Comparison of a user-centered design, self-management app to existing mhealth apps for persons living with HIV. JMIR Mhealth Uhealth.

[14] Schnall R, Rojas M, Bakken S, Brown W, Carballo-Dieguez A, Carry M, Gelaude D, Mosley JP, Travers J (2016). A user-centered model for designing consumer mobile health (mHealth) applications (apps). J Biomed Inform.

[15] Yang G, Long J, Luo D, Xiao S, Kaminga AC (2019). The characteristics and quality of mobile phone apps targeted at men who have sex with men in China: a window of opportunity for health information dissemination?. JMIR Mhealth Uhealth.

[16] Beach LB, Greene GJ, Lindeman P, Johnson AK, Adames CN, Thomann M, Washington PC, Phillips G (2018). Barriers and facilitators to seeking HIV services in Chicago among young men who have sex with men: perspectives of HIV service providers. AIDS Patient Care STDS.

[17] Catalani C, Philbrick W, Fraser H, Mechael P, Israelski DM (2013). mHealth for HIV treatment and prevention: a systematic review of the literature. Open AIDS J.

[18] Devi BR, Syed-Abdul S, Kumar A, Iqbal U, Nguyen P, Li YJ, Jian W (2015). mHealth: an updated systematic review with a focus on HIV/AIDS and tuberculosis long term management using mobile phones. Comput Methods Programs Biomed.

[19] Rossman K, Salamanca P, Macapagal K (2017). A qualitative study examining young adults' experiences of disclosure and nondisclosure of LGBTQ identity to health care providers. J Homosex.

[20] Mokgatle MM, Madiba S (2017). High acceptability of HIV self-testing among technical vocational education and training college students in Gauteng and North West province: what are the implications for the scale up in South Africa?. PLoS One.

[21] Majam M, Quaife M, Phatsoane M, Rhagnath N, Quaife M (2019). High self-reporting of HIV self-test results through an interactive voice response telephone line in inner city Johannesburg. Poster published in IAS Conference on HIV Science.

[22] De Boni RB, Lentini N, Santelli AC, Barbosa A, Cruz M, Bingham T, Cota V, Correa RG, Veloso VG, Grinsztejn B (2018). Self-testing, communication and information technology to promote HIV diagnosis among young gay and other men who have sex with men (MSM) in Brazil. J Int AIDS Soc.

[23] Janssen R, Engel N, Esmail A, Oelofse S, Krumeich A, Dheda K, Pai NP (2020). Alone but supported: a qualitative study of an HIV self-testing app in an observational cohort study in South Africa. AIDS Behav.

[24] Pant Pai N, Smallwood M, Desjardins L, Goyette A, Birkas KG, Vassal A, Joseph L, Thomas R (2018). An unsupervised smart app-optimized HIV self-testing program in Montreal, Canada: cross-sectional study. J Med Internet Res.

[25] Chan PS, Chidgey A, Lau J, Ip M, Lau JT, Wang Z (2021). Effectiveness of a Novel HIV self-testing service with online real-time counseling support (HIVST-online) in increasing HIV testing rate and repeated HIV testing among men who have sex with men in Hong Kong: results of a pilot implementation project. Int J Environ Res Public Health.

[26] Witzel TC, Gabriel MM, McCabe L, Weatherburn P, Gafos M, Speakman A, Pebody R, Burns FM, Bonell C, Lampe FC, Dunn DT, Ward D, Harbottle J, Phillips AN, McCormack S, Rodger AJ (2019). Pilot phase of an internet-based RCT of HIVST targeting MSM and transgender people in England and Wales: advertising strategies and acceptability of the intervention. BMC Infect Dis.

[27] Sanabria G, Scherr T, Garofalo R, Kuhns LM, Bushover B, Nash N, Davis R, Schnall R (2021). Usability evaluation of the mLab app for improving home HIV testing behaviors in youth at risk of HIV infection. AIDS Educ Prev.

[28] Gous N, Fischer AE, Rhagnath N, Phatsoane M, Majam M, Lalla-Edward ST (2020). Evaluation of a mobile application to support HIV self-testing in Johannesburg, South Africa. South Afr J HIV Med.

[29] Thonon F, Fahmi S, Rousset-Torrente O, Bessonneau P, Griffith JW, Brown C, Chassany O, Duracinsky M (2021). Promoting HIV, Hepatitis B virus, and Hepatitis C virus screening among migrants with a language barrier: protocol for the development and evaluation of an electronic app (Apidé). JMIR Res Protoc.

[30] Itoh N, Mishima H, Yoshida Y, Yoshida M, Oka H, Matsudaira K (2022). Evaluation of the effect of patient education and strengthening exercise therapy using a mobile messaging app on work productivity in Japanese patients with chronic low back pain: open-label, randomized, parallel-group trial. JMIR Mhealth Uhealth.

[31] Ellmann S, Maryschok M, Schöffski O, Emmert M (2022). The German COVID-19 digital contact tracing app: a socioeconomic evaluation. Int J Environ Res Public Health.

[32] Linardon J, King T, Shatte A, Fuller-Tyszkiewicz M (2022). Usability evaluation of a cognitive-behavioral app-based intervention for binge eating and related psychopathology: a qualitative study. Behav Modif.

[33] Schepens Niemiec SL, Wagas R, Vigen CL, Blanchard J, Barber SJ, Schoenhals A (2022). Preliminary user evaluation of a physical activity smartphone app for older adults. Health Policy Technol.

[34] Luna-Perejon F, Malwade S, Styliadis C, Civit J, Cascado-Caballero D, Konstantinidis E, Abdul SS, Bamidis PD, Civit A, Li Y( (2019). Evaluation of user satisfaction and usability of a mobile app for smoking cessation. Comput Methods Programs Biomed.

[35] Zelmer J, van Hoof K, Notarianni M, van Mierlo T, Schellenberg M, Tannenbaum C (2018). An assessment framework for e-mental health apps in Canada: results of a modified Delphi process. JMIR Mhealth Uhealth.

[36] Manning JB, Blandford A, Edbrooke-Childs J (2022). Digital companion choice to support teachers' stress self-management: systematic approach through taxonomy creation. JMIR Form Res.

[37] Sun X, Lu F, Wu Z, Poundstone K, Zeng G, Xu P, Zhang D, Liu K, Liau A (2010). Evolution of information-driven HIV/AIDS policies in China. Int J Epidemiol.

[38] Nour M, Chen J, Allman-Farinelli M (2019). Young adults' engagement with a self-monitoring app for vegetable intake and the impact of social media and gamification: feasibility study. JMIR Form Res.

[39] Gillette E, Naanyu V, Nyandiko W, Chory A, Scanlon M, Aluoch J, Koros H, Ashimosi C, Beigon W, Munyoro D, Lidweye J, Nyagaya J, DeLong A, Kantor R, Vreeman R (2023). HIV-related stigma shapes research participation for youth living with HIV in Kenya. J Int Assoc Provid AIDS Care.

[40] Khati A, Wickersham JA, Rosen AO, Luces JR, Copenhaver N, Jeri-Wahrhaftig A, Ab Halim MA, Azwa I, Gautam K, Ooi KH, Shrestha R (2022). Ethical issues in the use of smartphone apps for HIV prevention in Malaysia: focus group study with men who have sex with men. JMIR Form Res.

[41] Bui TM, Hoang MT, Ngo TV, Do CD, Nghiem SH, Byrnes J, Phung DT, Nguyen TH, Vu GT, Do HT, Latkin CA, Ho RC, Ho CS (2021). Smartphone use and willingness to pay for HIV treatment-assisted smartphone applications among HIV-positive patients in urban clinics of Vietnam. Int J Environ Res Public Health.

[42] Kahnbach L, Lehr D, Brandenburger J, Mallwitz T, Jent S, Hannibal S, Funk B, Janneck M (2021). Quality and adoption of COVID-19 tracing apps and recommendations for development: systematic interdisciplinary review of European apps. J Med Internet Res.

[43] Fan Y, Wang Z, Deng S, Lv H, Wang F (2022). The function and quality of individual epidemic prevention and control apps during the COVID-19 pandemic: a systematic review of Chinese apps. Int J Med Inform.

[44] Mastro TD, Kim AA, Hallett T, Rehle T, Welte A, Laeyendecker O, Oluoch T, Garcia-Calleja JM (2010). Estimating HIV incidence in populations using tests for recent infection: issues, challenges and the way forward. J HIV AIDS Surveill Epidemiol.

